# IgH gene rearrangements as plasma biomarkers in Non-Hodgkin's Lymphoma patients

**DOI:** 10.18632/oncotarget.235

**Published:** 2011-03-08

**Authors:** Jian He, Jian Wu, Yuchen Jiao, Nina Wagner-Johnston, Richard F. Ambinder, Luis A Diaz, Kenneth W Kinzler, Bert Vogelstein, Nickolas Papadopoulos

**Affiliations:** ^1^ The Ludwig Center for Cancer Genetics and Therapeutics and Howard Hughes Medical Institute, Johns Hopkins Kimmel Cancer Center, The Johns Hopkins Medical Institutions, Baltimore, MD; ^2^ Washington University School of Medicine, St. Luis, MO; ^3^ The Ludwig Center for Cancer Genetics and Therapeutics and Howard Hughes Medical Institute, Johns Hopkins Kimmel Cancer Center, The Johns Hopkins Medical Institutions, Baltimore, MD

**Keywords:** Cancer, leukemia, lymphoma, biomarkers, gene rearrangements

## Abstract

New biomarkers with improved accuracy could be helpful for monitoring disease in patients with Non-Hodgkin's lymphomas (NHL). Towards this end, we have explored the feasibility of identifying the sequence of rearranged IgH genes using next-generation sequencing, then using PCR to detect specific rearranged DNA fragments in patients' plasma. By capturing and sequencing the IgH genomic regions (IgCap), we were able to detect and precisely determine the sequence of rearranged IgH loci in the tumors of three NHL patients. Moreover, circulating rearranged DNA fragments could be identified in the plasma of all three patients. Even in cases wherein tumor biopsies were unavailable, we were able to use the IgH capture approach to identify rearranged DNA loci in the plasma of 8 of 14 patients. IgCap may enable a more informed management of selected patients with NHL and other B-cell malignancies in the future.

## INTRODUCTION

As cancer chemotherapeutics improve, the need for companion diagnostics to monitor the effects of such therapeutics becomes progressively more important.[1] The ideal marker would be one that can be simply assessed without the need for repeat biopsies or exposure to irradiation, is absolutely specific for the presence of the tumor (to avoid false positives), is sensitive for the presence of disease, and is cost-effective.[2] Among the many new biomarkers being developed, those employing free, circulating somatically mutated DNA sequences in the plasma are particularly attractive because they can in theory meet all these criteria.[3, 4] In particular, mutations are exquisitely tumor-specific because they are not found in any collection of normal cells in the patient, and thereby have advantages over markers that are simply associated with tumors, such as CEA or PSA.[5]

Circulating mutant DNA has been found in a variety of solid tumors and initial studies have shown them to provide sensitivity and specificity comparable or superior to conventional disease indicators.[6] In liquid tumors such as leukemias, consistently fused genes like BCR-ABL provide extraordinarily useful markers for following patients during their treatment.[7] In leukemia patients, rearrangements can be accessed in the blood or bone marrow by virtue of the fact that any residual cancer cells will reside in these compartments.[8] In tumors such as lymphomas, however, circulating cells are not consistently found in patients' blood or marrow. Based on the above-cited results on solid tumors, however, we hypothesized that somatically rearranged DNA templates from lymphomas might be found in the cell-free fraction of blood, i.e., the plasma.

To test this hypothesis, and to develop a generally applicable tool for companion diagnostics of B-cell lymphomas, it was first necessary to identify aberrant DNA sequences that could be identified in lymphoma patients. Though no specific point mutations or oncogene rearrangements are found in lymphomas in general, virtually all lymphomas harbor rearrangements in their immunoglobulin (Ig) genes.[9] Clever assays that reveal such rearrangements have been described, particularly those employing multiplex PCR to identify clonal rearrangements upon electrophoresis through an increase in the signal of a fragment “spike” representing the rearrangement.[10] Though such assays are clinically useful in many situations, the “spikes” representing clonal rearrangements can be difficult to detect, either because of inefficient primer annealing to the hypermutated sequences in rearranged Ig genes. More importantly, such assays cannot provide optimal sensitivity, as the tumor-specific spikes are overlaid on a background of normal Ig rearrangements. The tumor-specific rearrangements can therefore only be observed if their abundance is significantly greater than the aggregate level of rearrangements from normal B cells. Nonetheless, the appearance of such spikes in plasma correlates with the presence of lymphoma and persistence of such spikes following chemotherapy seems to portend a poor prognosis. To enhance the specificity of this plasma-based approach, we set out to use a capture-and-sequence method to more specifically identify rearrangements of IgH genes at the sequence level then to use this information to detect the same rearrangements in the plasma of NHL patients.

## RESULTS

### Strategy

We chose to identify the rearrangements in DNA rather than RNA because RNA of adequate quality is not available in many clinical situations while DNA can be readily obtained even from archival samples. IgCap involves three steps: (i) the tumor DNA is first randomly sheared and ligated to adapters that allow their subsequent amplification by PCR; (ii) fragments containing IgH genes are captured on a solid support containing the sequences of interest [11]; and (iii) the captured DNA is amplified by conventional PCR, producing an IgCap library, and the ends of the captured DNA fragments are subjected to massively parallel sequencing.

The sequence information obtained is then processed *in silico* to identify rearranged sequences. Note that IgCap captures *all* fragments containing relevant IgH gene sequences, not simply the rearranged fragments. Moreover, the actual targets of this analysis, i.e., the rearranged loci, are nearly always mutated, both within the exons and at the borders of the rearranged exons. Both these features make the identification of rearrangements challenging, particularly when only a relatively small number of bases from each fragment are determined, as with the Illumina instrument. However, we were able to develop algorithms that could identify rearranged IgH genes on the basis of several features that distinguish them from unrearranged genes. In brief, we developed two algorithms, one that could be used for analysis of one end of a tag and the other for both ends in paired-end reads. The first algorithm (called the “CTGGGG-algorithm) identified “seed” fragments that contained a 6 nt sequence which was identical to, or differed at one position, from a conserved sequence present in all J genes (CTGGGG). The second algorithm (called the “paired-end algorithm”), used paired-end reads to identify two sets of seed fragments. The first set included fragments related to normal V, D or J regions but whose ends represented sequences separated by >10000 bp in unrearranged DNA and in the expected orientation. The second set included fragments in which one of the two ends was related to normal V regions and the other end included specific sequences within J or D regions. The J-specific sequence was CTGGGCCA, while the D-specific sequences included the middle five bases of each of the D regions. In both algorithms, seed fragments were extended to include larger regions of V, D or J by performing homology searches among the other fragments in the sequenced IgCap library.

To determine the sensitivity of these algorithms for identifying rearranged IgH genes, we tested it on 89 known rearrangements recorded in the IMGT/LIGM-DB database (Supplementary Table 1). We randomly cleaved ~100,00 bases spanning each rearrangement *in silico* to generate a virtual library of overlapping fragments of 100 bp that mimicked the size of the DNA fragments actually used to make IgCap libraries. Each library was then analyzed using the two algorithms. The combination of the alogorithms resulted in the identification of all 89 re-arrangements. No rearrangements were detected in analogous *in silico* libraries constructed from unrearranged IgH loci.

### Identification of rearranged IgH genes directly from plasma

**Figure 1:**
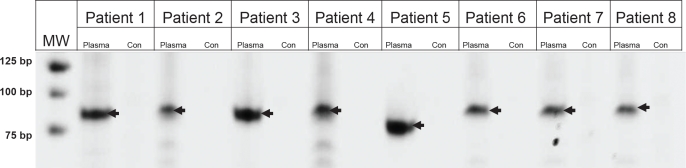
PCR amplification of Ig rearrangements in plasma DNA was purified from either the plasma of patients or from normal cells of an unrelated patient (Con). MW, molecular weight markers, size indicated on left in base pairs (bp).

Using the approach described above, we first attempted to directly identify IgH gene rearrangements in DNA purified from plasma in 14 patients in whom no tumor tissue was available (clinical characteristics are described in Supplementary Table 2). We used an Illumina GA2 sequencer to analyze one end of each tag, employing one lane per patient. We were thereby able to generate 2,673,399 to 18,889,095 tags of high quality from the 14 patients (Table 1). In ten of the samples, we identified putative rearrangements (Table 2). We applied the same procedure to the plasma DNA of two individuals without B-cell neoplasia, and did not identify any rearrangements. We then designed primer pairs (Supplementary Table 3) that straddled the ten NHL patient rearrangements and used them to PCR-amplify DNA from the same plasma samples. In eight of the ten cases, we identified PCR products of the expected size in the plasma of the appropriate patients but not in DNA from normal individuals (Figure 1). The PCR fragments were excised from the gel, cloned, and sequenced. In each case, the sequence was that predicted from the algorithm (with the exception of a single base substitution that could have arisen during cloning, whole genome amplification or clonal progression).

**Table 1:** Sequencing summary

Patient ID	Total tags	Bases Sequenced	Tags matched uniquely to human genome	Bases matched to genome	Tags matched to the Ig region	Tags matched to the Ig coding sequences	Bases matched to the Ig region	Average coverage of the Ig region	Target bases with more than 10 reads (number)	Target bases with more than 10 reads (%)
Patient 1	18,889,095	944,454,750	7,359,299	367,964,950	214,548	88,916	10,727,400	251	14437	61%
Patient 2	3,183,730	159,186,500	1,109,460	55,473,000	166,626	97,334	8,331,300	275	13826	59%
Patient 3	11,265,224	563,261,200	6,077,027	303,851,350	164,494	78,858	8,224,700	223	14373	61%
Patient 4	17,306,516	865,325,800	5,053,458	252,672,900	320,330	162,534	16,016,500	459	14401	61%
Patient 5	11,149,375	557,468,750	4,385,951	219,297,550	114,142	52,816	5,707,100	149	13912	59%
Patient 6	4,166,839	208,341,950	2,351,275	117,563,750	119,952	59,362	5,997,600	168	14588	62%
Patient 7	3,875,106	193,755,300	2,405,508	120,275,400	125,808	64,365	6,290,400	182	14611	62%
Patient 8	9,741,614	487,080,700	6,221,945	311,097,250	131,132	57,886	6,556,600	163	14726	62%
Patient 9	2,673,399	133,669,950	1,391,649	69,582,450	36,284	19,443	1,814,200	55	13541	57%
Patient 10	16,909,387	845,469,350	10,206,055	510,302,750	156,254	70,047	7,812,700	198	14656	62%
Patient 11	15,218,254	760,912,700	7,460,806	373,040,300	450,949	268,217	22,547,450	757	15888	67%
Patient 12	9,693,373	484,668,650	5,592,951	279,647,550	145,140	64,939	7,257,000	183	14526	62%
Patient 13	3,344,057	167,202,850	1,982,627	99,131,350	53,919	31,062	2,695,950	88	13644	58%
Patient 14	16,818,309	840,915,450	6,464,306	323,215,300	334,392	155,854	16,719,600	440	14642	62%
Patient 15	22,785,641	1,139,282,050	12,265,838	613,291,900	830,469	609,774	41,523,450	1721	16851	95%
Patient 16	19,547,977	977,398,850	11,370,485	568,524,250	789,493	586,682	39,474,650	1656	16866	95%
Patient 17	21,925,769	1,096,288,450	12,059,079	602,953,950	701,194	503,556	35,059,700	1421	16837	95%

**Table 2:** Rearrangements identified in this study

Patient ID	Sample type	Rearranged Sequence Identified	Rearranged Genes	Validated by PCR*
Patient 1	Plasma	CTGGGTGGATTCTGAACAGCCCCGAGTCACGGTGGGTATAGTGGGAGCCGAGGCCTACTGGGGCCAGGGAACCCTGGTCACCGTCTCCTCAGGTGAGTCCT	IGHD1-1:J5	Yes
Patient 2	Plasma	GCAGACACGGCTGTGTATTACTGTGCGAGACTGGGATCCCCGTATAGCAGCAGCTGGCCCTACTACTACGGTATGGACGTCTGGGGCCAAGGGACCACGGTCACCGTCTC	IGHV4-30-2:J6	Yes
Patient 3	Plasma	CCAGCCCCCAGGGAAGGGACTGGAGTGGATTGGGAGTGTTGATTATTCTGGGGACACCTTCCATAACCCATCCCTCAAGAGTCGCGTCTCCATATTAATAGACGCGTCTAAAAACGTTTTCTCTCTGAGGTTGACTTTTGTTACCGCCGCGGACACGGCCATATATTATTGTGCGGGACATCCTCATAGTACTGGGTGGTATCAATCTGGGAACTGGTTCGACTCCTGGGGCCAGGGAACCCCGGTCACCGTCTCCTCA	IGHV4-4:J5	Yes
Patient 4	Plasma	CCGCGGACACGGCCATCTATTACTGTGCGAGAGATAAAGCAGATAAAACATTACGATGGCCCAAGACCTTAAACTGGTTCGACCCCTGGGGCCACGGAACCCTGGTCACC	IGHD4-23:J5	Yes
Patient 5	Plasma	CCTGTGGACACAGCCACATATTACTGTGCAGCAGTAGAACTAATTGAGGCCGGCATGGAACTTGGCTACTGGGGCCCCCGGGAATCCTGGTCAACGTCTCCTCAGGTGAGTCCTC	IGHV2-5:J5	Yes
Patient 6	Plasma	TGTCACTGTGGTATTACGATTCTTTGACTGGTTGTTGTCAGCGCGACCCCTCTTTGACTACTGGGGCCAGGGAACCCTGGTCACCGTCTCCTCAGGTGA	IGHD3-9:J4	Yes
Patient 7	Plasma	CCAGGTGTGGTTATTGTCAGGGGGTGCCAGGCCGTGGTAGAGATGGCTACAACTACCAACCACGAGGGTACTACTTTGACTACTGGGGCCAGGGAACCCTGGTCACC	IGHD5-24:J4	Yes
Patient 8	Plasma	CAGGCCGTGGTAGAGATGGCTACAATCAGGATTGCATGGTCGTCGTTTACGGGGGGTTTTCTACTGGGGCCAGGGAACCCTGGTCACCGTCTC	IGHD5-24:J4	Yes
Patient 9	Plasma	TGTGGTATTACTATGATAGTAGTGTGAGGGGCCAGGGAACCCTGGTCACCGTCTCCTCA	IGHD3-22:J5	No
Patient 10	Plasma	AACCGAGGACACAGCCGTGTATTACTGTACCACAGGGGCGTACTGGGGCCAGGGAACCCT	IGHV3-49:J4	No
Patient 11	Plasma	-	-	No
Patient 12	Plasma	-	-	No
Patient 13	Plasma	-	-	No
Patient 14	Plasma	-	-	No
Patient 15	Primary tumor	TCTGTGACCGCCGCAGACACGGCTGTGTATTTTTGTGCGAGGCAGGTTGCGACGCAGGGGGCCCCCTTTGACTTCTGGGGCCGGGGAACCCTGATCACCGTCTCCTCAGGTGAGTCCT	IGHV4-30-2:J4	Yes
Patient 15	Primary tumor	TGAGGTCTGTGTCACTGTGATATTATGATACTTTGACTGGTTATTTGAGAAAGTTGACTACTGGGGCCAGGGAACCCTGGTCACCGTCTCCTCAGG	IGHD3-9:J4	Yes
Patient 16	Primary tumor	GTACGTGCCATGTTAGGATTTTGATCGAGGAGACAGCACCATGGGTATGGTTTCACTGGGGCCAGGGCACCCTGGTCACCGTCTCCTCAGGTGA	IGHD3-10:J1	Yes
Patient 16*	Primary tumor	TTCCAGGTGTGGTTATTGTCAGGCGATGTCAGACTGTGGTGGATATAGTGGCTACGATCCCTCACCTTCTGGGGCCAAGGGACAATGGTCACCGTCTCTTCAGGTAAGATGGCT	IGHD5-12:J3	Yes
Patient 17	Primary tumor	GTAGGGACAGGAGGATTTTGTGGGGGCTCGAGTCACTGTGAGCATATTGTGGTGGTGATTGCTCCGCTGAATACTTCCACCACTGGGGCCAGGGCACCCTGGTCACCGTCTCC	IGHD2-21:J1	Yes
Patient 17	Primary tumor	TCTTCAAATGAACAACCTGAGAGTCGAGGACACGGCCGTTTATTACTGTACGAGAGAGACAAATTGTGACTACTGGGGCCAGGGAACCCTGGTCACCGTCTCCTCAGGTGA	IGHV3-53:J5	Yes
* The sequence identified through analys of the IgCap libraries was identical to that determined from analysis of the PCR products used for validation except in Patient 16, in which the sequence differed by a single base (G > A) at position 25.				

### Identification of rearranged IgH genes in tumors

Though the results described above were encouraging, we could not definitively identify rearrangements in 6 of the 14 patients. Moreover, the number of IgCap library fragments containing sequences corresponding to the rearranged gene was generally small, ranging from 1 to 11 in those patients in which a rearrangement could be identified. Plasma DNA is certainly not enriched in rearranged fragments, and most IgH gene-containing DNA fragments from plasma are undoubtedly derived from non-tumor tissue. To improve the sensitivity of IgCap, two modifications were made. First and most importantly, we used a portion of the original lymphoma, rather than the plasma, to identify rearrangements, then used plasma to determine whether the rearranged IgH gene could be detected in the circulation. Second, it became possible to sequence both ends of library fragments (“paired-end reads”) during the course of this study, and this sequencing approach was employed in the remaining experiments.

This modified strategy was applied to DNA isolated from the involved lymph nodes of three NHL patients (clinical information in Supplementary Table 2). From a single lane of an Illumina GAIIx sequencer, we recovered 20 million tags of high sequence quality, 6-7% of which contained homology to V, D, or J regions (Table 1). In each of the three patients, IgCap identified two rearrangements that were represented by more than 8 tags (Table 2). One of the two rearrangements in each patient was presumably derived from the maternal allele and the other from the paternal allele. Importantly, the number of IgCap library fragments containing sequences that bridged the rearrangement was more than one in each case, ranging from 8 to 36. In two of the six rearrangements, we could identify both VD and DJ junctions. In the remaining 4, we could only identify the DJ junction. We did not determine whether this was due to technical vs. biologic reasons; it is well-known that incomplete rearrangements, in which D is fused to J but V is not fused to D, occur in neoplastic B-cells.[12]

**Figure 2:**
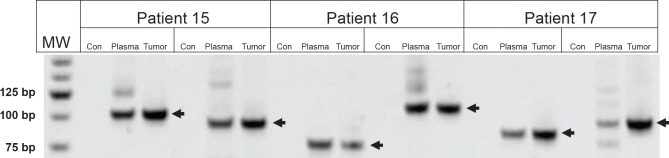
PCR amplification of Ig rearrangements in tumors and plasma Two Ig rearrangements are shown for each patient sample. DNA was purified from either from the tumor and plasma of patients or from normal cells of an unrelated patient (Con). MW, molecular weight markers, size indicated on left in base pairs (bp).

PCR primers that straddled the six rearrangements were used to amplify DNA from the three tumors. In each case, PCR products of the expected size were found in the tumors of the appropriate patients but not in germ-line DNA from normal individuals (Figure 2). The PCR fragments were excised from the gel, cloned, and sequenced. In each case, the sequence was that predicted from the IgCap data.

We next attempted to determine whether the rearranged fragments could be identified in the plasma of these three patients. In all cases, both rearranged fragments were evident and could be detected in as little as 30 ul of plasma (Figure 2). The PCR fragments from plasma were cloned and found to be identical in sequence to those of the corresponding patients' tumors. The number of rearranged fragments was determined by digital PCR and found to vary from 30 to 100 fragments per ml of plasma. These rearrangements were not detected in DNA from circulating cells from the same patients, even when the DNA from as many as 1 million circulating cells were assessed. . These results are consistent with the idea that lymphoma cells are rare in the circulation and that the rearranged DNA is released from the tumors in situ rather than derived from circulating lymphoma cells.

## DISCUSSION

The results described above show that rearranged IgH genes can be routinely identified in NHL tumors, that the rearranged genes are present in the circulation at detectable levels, and that the circulating DNA is more abundant than circulating lymphoma cells. Moreover, the IgCap approach is powerful enough to identify some patients' rearrangements directly from plasma if no tumor biopsy is available. These results set the stage for clinical implementation of this type of biomarker to follow the course of NHL patients following therapy.

The clonal assessment of rearrangements in immunoglobulin or T cell receptor genes has a long history.[10] Initially, Southern blotting was the gold standard for this type of analysis, but required large amounts of high quality genomic DNA and lacked the sensitivity required for many types of diagnostic assays. Southern blotting was replaced with PCR-based assays, which have many advantages. Primer sets facilitating multiplex PCR assays have been painstakingly designed and shown to detect up to 74% DLBCL and 96% FL.[13] Massively parallel sequencing of PCR products derived from such primer sets has recently been used to identify VDJ rearrangements in Ig or T-cell receptor genes. [14-16] The massively parallel sequencing approach used herein, in which VDJ genes are captured rather than amplified using Ig-specific primers, represents another means towards the identification of clonal rearrangements and their use as biomarkers. Though more complex than the direct PCR approach, it has the advantage of being less biased; in theory, it should detect 100% of rearranged fragments from lymphomatous tissues. Additionally, it can be applied to degraded DNA samples such as those from paraffin-embedded material or plasma. Degraded DNA cannot efficiently be amplified with conventionally used primer sets because the product size is larger than the size of the DNA.

We used two algorithms to identify rearranged Igs. The CTGGGG-based algorithm detects all typical rearranged Igs. However, this algorithm can fail to detect uncommon Ig rearrangements such as those involving only V and D or those involving J region deletions. The paired-end algorithm is complementary. It has the capacity to pick out any rearrangement involving the IgH locus, including Ig rearrangements as well as translocations such as t(8;14)(q24;q32) in Burkitt lymphoma and t(11;14)(q13;q32) in mantle cell lymphoma.

As with all biomarkers, the approach described here has limitations. Its major conceptual limitation revolves around marker stability: a relapsed lymphoma may have undergone clonal divergence so that its rearrangement would be undetectable upon PCR with the primers used to validate the original tumor.[17] It was therefore comforting that in each of the three NHL tumor samples evaluated in this study, two distinct rearrangements were identified, and both could be identified in the plasma of each patient. The availability of two independent markers reduces the probability of a false negative during follow-up. Because the exact sequence of the rearrangement is determined with our approach, two sets of primers for each rearrangement could be employed, thereby further minimizing the potential impact of clonal evolution within the rearranged loci.

Enthusiasm has been growing for risk-adapted therapy in the treatment of diffuse large B cell lymphoma. Mid cycle FDG PET has been used to guide decisions on switching chemotherapy regimens and to select patients for high dose consolidation.[18],[19] However, standardized interpretation of PET imaging still presents obstacles, as recently noted in an ongoing cooperative group trial.[20] The plasma-based approach described here represents an alternative and perhaps complementary approach to tumor assessment. Another important issue to be addressed in the future is the sensitivity of the method. In this work, we have only evaluated patients with clinically apparent disease. It may be more difficult to detect rearranged IgH genes in the plasma of patients with minimal residual disease following therapy. However, we were encouraged by the fact that rearrangements can be detected in as little as 30 ul of plasma in the three patients whose levels were quantified. This suggests that disease burdens amounting to only 1% of those of the studied patients could be readily detected in 3 ml of plasma, assuming the relationship between disease burden and plasma DNA is linear.

Do the rearrangements detected with IgCap represent those occurring in the tumor cells? In the three cases in which the rearrangements were identified in the tumor, there can be little doubt of this, as the number of IgCap library fragments corresponding to the major rearrangement outnumbered any other putative rearrangements in the sample by more than a hundred-fold. In the cases in which only plasma DNA was available, it is theoretically possible that the identified rearrangements arose from normal B-cells. Two observations argue against this possibility. First, we have attempted the IgCap strategy on two samples of plasma from patients without B-cell neoplasms and have not identified any rearranged fragments in IgCap libraries from them. Second, there were two patients in whom a plasma sample taken during complete remission was available, and the rearrangements could not be identified in these either (through PCR with the rearrangement-specific primers). Regardless, it is clearly optimal to identify the rearranged biomarker by employing the IgCap strategy on DNA from lymphomas, rather than from plasma, whenever the former is available. The biomarker can then be easily applied to plasma using PCR, as described above.

In sum, IgCap is an advanced, personalized medicine approach that provides exciting research opportunities and offers the potential for clinical application. The basic idea of IgCap could be applied to other diseases, including those with an autoimmune or allergic basis, to define the number and nature of Ig rearrangements in clinical samples. With minor modifications, it might also be applied to the evaluation of T-cells.

## MATERIALS AND METHODS

### Samples

All samples in this study were obtained from patients under an Institutional Review Board protocol.

### DNA purification

DNA was extracted from 1000 μL of plasma with a QIAamp Circulating Nucleic Acid kit following the manufacturer's instructions. (QIAGEN; Valencia, CA). DNA was extracted from tumor tissues and normal cells using a Qiagen AllPrep kit following the manufacturer's protocol.

### Illumina library preparation

Tumor genomic DNA libraries were prepared following Illumina's (Illumina, San Diego, CA) protocol with the following modifications. (1) Three micrograms (μg) of genomic DNA in 100 microliter (μl) of TE was fragmented in a Covaris sonicator (Covaris, Woburn, MA) to a size of 100-500bp. DNA was purified with a PCR purification kit (Cat # 28104, Qiagen, Valencia, CA) and eluted in 35μl of elution buffer included in the kit. (2) Purified, fragmented DNA was mixed with 40 μl of H_2_O, 10 μl of 10xT4 ligase buffer with 10mM ATP, 4 μl of 10mM dNTP, 5 μl of T4 DNA polymerase, 1 μl of Klenow Polymerase, and 5 μl of T4 polynucleotide Kinase. All reagents used for this step and those described below were from New England Biolabs (NEB, Ipswich, MA) unless otherwise specified. The 100μl end-repair mixture was incubated at 20°C for 30 min, purified by a PCR purification kit (Cat # 28104, Qiagen) and eluted with 32μl of elution buffer (EB). (3) To A-tail, all 32 μl of end-repaired DNA was mixed with 5 μl of 10xBuffer (NEB buffer 2), 10 μl of 1mM dATP and 3 μl of Klenow (exo-). The 50 μl mixture was incubated at 37°C for 30 min before DNA was purified with a MinElute PCR purification kit (Cat # 28004, Qiagen). Purified DNA was eluted with 12.5 μl of 70°C EB. (4) For adaptor ligation, 10 μl of A-tailed DNA was mixed with 10 μl of adapters (Illumina), 25 μl of 2x Rapid Lgase buffer and 5 μl of Rapid Ligase. The ligation mixture was incubated at room temperature (RT) for 15 min. (5) To purify adapter- ligated DNA, 50 μl of ligation mixture from step (4) was mixed with 200 μl of NT buffer from NucleoSpin Extract II kit (cat# 636972, Clontech, Mountain View, CA) and loaded into a NucleoSpin column. The column was centrifuged at 13,000 g in a desktop centrifuge for 1 min, washed once with 600 μl of wash buffer (NT3 from Clontech), and centrifuged again for 2 min to dry completely. DNA was eluted in 50μl elution buffer included in the kit. (6) To obtain an amplified library, ten PCRs of 25 μl each were set up, each including 12 μl of H_2_O, 5 μl of 5 x Phusion HF buffer, 0.5 μl of 10 mM dNTP, 1.25 μl of DMSO, 0.5 μl of Illumina PE primer #1, 0.5 μl of Illumina PE primer #2, 0.25 μl of Hotstart Phusion polymerase, and 5 μl of the DNA from step (5). The PCR program used was: 98°C 1 minute; 6 cycles of 98°C for 20 seconds, 65°C for 30 seconds, 72°C for 30 seconds; and 72°C for 5 min. To purify the PCR product, 250 μl PCR mixture (from the ten PCR reactions) was mixed with 500 μl NT buffer from NucleoSpin Extract II kit and purified as described in step (5). Library DNA was eluted with elution buffer pre-heated to 70°C and the DNA concentration was estimated by absorption at 260 nm.

### IgCap capture

The targeted capture region included V-gene exons plus the first 36 bp of the downstream introns, six J-gene exons plus the first 36 bp of the upstream introns, and all the D-gene exons. We obtained these regions through PCR of normal genomic DNA using the primers described in Supplementary Table 4 or custom synthesized probes and then used a strategy based on that described in [11] to capture the IgH genome. PCR using these primers was performed using the reaction conditions specified previously.[21]

### Confirmation of IgH rearrangements in tumor and plasma samples

The full V-D-J or D-J joint region sequence and 40 bp from either side of the joint were used for primer design. The PCR mixture (50 μl) contained various amounts of template DNA, 0.2 μM of forward-reverse primer mixture, 2.5 mM of MgCl2, 100 μM of dNTPs, and 0.5 units of Invitrogen Taq polymerase. PCR was performed as follows: 96°C for 4 min; 45 cycles of 96°C for 10 s, 59°C for 10 s, and 72°C for 30 s. The primers used for PCR are listed in Supplementary table 3. PCR products were gel purified with QIAquick *Gel Extraction Kit (*QIAGEN; Valencia, CA*) and* cloned with a TA clone kit (Promega, Madison, WI, USA) according to the manufacture's protocols. Plasmid DNA was evaluated by Sanger sequencing. Digital PCR was performed as described before.[22]

## SUPPLEMENTAL TABLE








