# Supplementary material for: IgH gene rearrangements as plasma biomarkers in Non-Hodgkin's Lymphoma patients
Source: Oncotarget. 2011 Mar 8;2(3):178–85. doi: 10.18632/oncotarget.235 (PMC3260819; doi:10.18632/oncotarget.235)
Supplement: Supplementary file 1 [file oncotarget-02-178-s001.docx]

Supplementary Table 1. List of rearranged Ig genes tested *in silico*.

Locus	Description
AF304552	Homo sapiens isolate 13-9 immunoglobulin heavy chain VH3 region gene, partial cds. ; genomic DNA; rearranged configuration; IgHeavy; regular; group IGHV.
[illegible]	Homo sapiens isolate [illegible] immunoglobulin heavy chain VH3 region gene, partial cds. ; genomic DNA; rearranged configuration; IgHeavy; regular; group IGHV.
[illegible]	Homo sapiens isolate [illegible] VH3 nonfunctional immunoglobulin heavy chain VH3 region gene, partial sequence. ; genomic DNA; rearranged configuration; IgHeavy; [illegible]
AF304555	Homo sapiens isolate 13-9 immunoglobulin heavy chain VH3 region gene, partial cds. ; genomic DNA; rearranged configuration; IgHeavy; regular; group IGHV.
AF306542	Homo sapiens clone STD_10 immunoglobulin heavy chain gene, partial cds. ; genomic DNA; rearranged configuration; IgHeavy; regular; group IGHV.
[illegible]	Homo sapiens clone [illegible] immunoglobulin heavy chain variable [illegible] gene, VH3 region ; genomic DNA; rearranged configuration; IgHeavy; regular [illegible]
[illegible]	Homo sapiens clone [illegible] immunoglobulin heavy chain variable region gene, partial cds. ; genomic DNA; rearranged configuration; IgHeavy; regular; group IGHV.
AF420028	Homo sapiens clone 3 nonfunctional immunoglobulin heavy chain variable region gene, partial sequence. ; genomic DNA; rearranged configuration; IgHeavy; regula[illegible]
AF442756	Homo sapiens isolate 204 immunoglobulin heavy chain variable region mRNA, partial cds. ; RNA; rearranged configuration; IgHeavy; regular; functionality productive[illegible]
[illegible]	Homo sapiens immunoglobulin heavy chain variable region gene, partial cds. ; genomic DNA; rearranged configuration; IgHeavy; regular; group IGHV.
[illegible]	Homo sapiens clone [illegible] immunoglobulin heavy chain variable region [illegible] gene, partial cds. ; genomic DNA; rearranged configuration; Ig[illegible]
AJ001942	Homo sapiens immunoglobulin M heavy chain variable region gene (cell PM1) ; genomic DNA; rearranged configuration; IgHeavy-Mc; regular; group IGHV.
AJ005975	Homo sapiens mRNA for immunoglobulin heavy chain clone ID-6-6 T ; genomic DNA; rearranged configuration; IgHeavy; regular; group IGHV.
[illegible]	Homo sapiens mRNA for variable region of IgHeavy chain, rearrangement of [illegible] ; genomic DNA; rearranged configuration; IgHeavy; regular; group IGHV.
[illegible]	Homo sapiens mRNA for immunoglobulin heavy chain variable region, clone [illegible] ; genomic DNA; rearranged configuration; IgHeavy; regular; group IGHV.
AJ222670	Homo sapiens mRNA for rearranged IgM heavy chain, IgVH4-21-D-JH6-Cmu, clone [illegible] ; genomic DNA; rearranged configuration; IgHeavy-Mc; group IGHV.
AJ239264	Homo sapiens mRNA for Ig heavy chain variable region, clone C9 ; genomic DNA; rearranged configuration; IgHeavy; regular; group IGHV.
AJ239264	Homo sapiens mRNA for Ig heavy chain variable region, clone C2 ; genomic DNA; rearranged configuration; IgHeavy; regular; group IGHV.
[illegible]	Homo sapiens mRNA for Ig heavy chain variable region, clone C[illegible] ; genomic DNA; rearranged configuration; IgHeavy; regular; group IGHV.
AJ239252	Homo sapiens mRNA for Ig heavy chain variable region, clone C2 ; genomic DNA; rearranged configuration; IgHeavy; regular; group IGHV.
AJ239263	Homo sapiens mRNA for Ig heavy chain variable region, clone [illegible] ; genomic DNA; rearranged configuration; IgHeavy; regular; group IGHV.
AJ239270	Homo sapiens mRNA for Ig heavy chain variable region, clone C2 ; genomic DNA; rearranged configuration; IgHeavy; regular; group IGHV.
[illegible]	Homo sapiens mRNA for Ig heavy chain variable region, clone C2[illegible] ; genomic DNA; rearranged configuration; IgHeavy; regular; group IGHV.
[illegible]	Homo sapiens mRNA for Ig heavy chain variable region, clone C1 ; genomic DNA; rearranged configuration; IgHeavy; regular; group IGHV.
AJ239284	Homo sapiens mRNA for Ig heavy chain variable region, clone C23 ; genomic DNA; rearranged configuration; IgHeavy; regular; group IGHV.
AJ239292	Homo sapiens mRNA for Ig heavy chain variable region, clone C5 ; genomic DNA; rearranged configuration; IgHeavy; regular; group IGHV.
[illegible]	Homo sapiens DNA for immunoglobulin heavy chain variable region, partial, clone [illegible] ; genomic DNA; rearranged configuration; IgHeavy; regular; group IGHV.
[illegible]	Homo sapiens DNA for immunoglobulin heavy chain variable region, partial, clone [illegible] ; genomic DNA; rearranged configuration; IgHeavy; regular; group IG[illegible]
AJ289253	Homo sapiens partial IGHV gene for immunoglobulin heavy chain variable region, clone A50 ; genomic DNA; rearranged configuration; IgHeavy; regular; group IGHV.
AJ289256	Homo sapiens partial IGHV gene for immunoglobulin heavy chain variable region, clone A06 ; genomic DNA; rearranged configuration; IgHeavy; regular; group IGHV.
[illegible]	Homo sapiens partial IGHV gene for immunoglobulin heavy chain variable region, clone [illegible] ; genomic DNA; rearranged configuration; IgHeavy; regular; group IGHV.
[illegible]	Homo sapiens mRNA for immunoglobulin M heavy chain variable region, partial clone [illegible] ; RNA; rearranged configuration; IgHeavy; regular; functionality [illegible]
AJ289283	Homo sapiens partial IGHV gene for immunoglobulin heavy chain variable region, clone A88 ; genomic DNA; rearranged configuration; IgHeavy; regular; group IGHV.
AJ300787	Homo sapiens partial mRNA for immunoglobulin gamma heavy chain variable region (IGHV3-30), clone [illegible] ([illegible]), Kawasaki disease patient ; RNA; rearrange[illegible]
[illegible]	Homo sapiens partial mRNA for immunoglobulin gamma heavy chain variable region (IGHV3-30), clone [illegible] ([illegible]), Kawasaki disease patient ; RNA; rearrang[illegible]
[illegible]	Homo sapiens partial IGHV gene for immunoglobulin heavy chain variable region, rearrangement [illegible] ; genomic DNA; rearranged configuration; IgHeavy; reg[illegible]
AJ305398	Homo sapiens partial IGHV gene for immunoglobulin heavy chain variable region, rearrangement [illegible] ; genomic DNA; rearranged configuration; IgHeavy; re[illegible]
AJ306872	Homo sapiens partial mRNA for anti-DNA immunoglobulin heavy chain variable region (IGHV-PAC3 gene) ; RNA; rearranged configuration; IgHeavy; regular; functio[illegible]
AJ306877	Homo sapiens partial mRNA for anti-DNA immunoglobulin heavy chain variable region (IGHV-PAC2 gene) ; RNA; rearranged configuration; IgHeavy; regular; functio[illegible]
[illegible]	Homo sapiens partial mRNA for anti-DNA immunoglobulin heavy chain variable region (IGHV-PAC2 gene) ; RNA; rearranged configuration; IgHeavy; regular; functio[illegible]
[illegible]	Homo sapiens partial VH1-[illegible] gene for immunoglobulin heavy chain variable region, from [illegible] lymphoma [illegible] ; genomic DNA; rearranged configuration; Ig[illegible]
AJ417597	Homo sapiens IGHV gene for immunoglobulin heavy chain variable region, psoriatic arthritis clone [illegible] ; genomic DNA; rearranged configuration; IgHeavy; regula[illegible]
AJ417620	Homo sapiens IGHV gene for immunoglobulin heavy chain variable region, psoriatic arthritis clone [illegible] ; genomic DNA; rearranged configuration; IgHeavy; regula[illegible]
[illegible]	Homo sapiens partial rearranged DNA for immunoglobulin heavy chain variable region, [illegible] segments, clone [illegible] ; genomic DNA; rearranged configuratio[illegible]
[illegible]	Homo sapiens partial rearranged DNA for immunoglobulin heavy chain variable region, [illegible] segments, clone [illegible] ; genomic DNA; rearranged configuratio[illegible]
AJ512004	Homo sapiens partial IGHV gene for immunoglobulin heavy chain, exon 1, clone 22_19 ; genomic DNA; rearranged configuration; IgHeavy; regular; group IGHV.
AJ512006	Homo sapiens partial IGHV gene for immunoglobulin heavy chain, exon 1, clone 22_20 ; genomic DNA; rearranged configuration; IgHeavy; regular; group IGHV.
[illegible]	Homo sapiens partial IGHV gene for immunoglobulin heavy chain, exon 1, clone 22_5 ; genomic DNA; rearranged configuration; IgHeavy; regular; group IGHV.
[illegible]	Homo sapiens partial IGHV pseudogene, exon 1, clone 22_5 ; genomic DNA; rearranged configuration; IgHeavy; regular; group IGHV.
AJ512017	Homo sapiens partial IGHV gene for immunoglobulin heavy chain, exon 1, clone 22_9 ; genomic DNA; rearranged configuration; IgHeavy; regular; group IGHV.
AJ627941	Homo sapiens partial IGHV3-48 gene for immunoglobulin heavy chain variable region, clone [illegible] ; genomic DNA; rearranged configuration; IgHeavy; regular; g[illegible]
[illegible]	Homo sapiens partial IGHV3-15 gene for immunoglobulin heavy chain variable region, clone [illegible] ; genomic DNA; rearranged configuration; IgHeavy; regular [illegible]
[illegible]	Homo sapiens partial IGHV4-34 gene for immunoglobulin heavy chain variable region, clone [illegible] ; genomic DNA; rearranged configuration; IgHeavy; regular [illegible]
AJ627963	Homo sapiens partial IGHV4-30-4 gene for immunoglobulin heavy chain variable region, clone [illegible] ; genomic DNA; rearranged configuration; IgHeavy; regul[illegible]
AJ627969	Homo sapiens partial IGHV4-30-2 gene for immunoglobulin heavy chain variable region, clone [illegible] ; genomic DNA; rearranged configuration; IgHeavy; regul[illegible]
AJ868226	Homo sapiens partial IGHV gene for immunoglobulin heavy chain variable region, clone [illegible] ; genomic DNA; rearranged configuration; IgHeavy; regular; functio[illegible]
[illegible]	Homo sapiens partial IGHV pseudogene, clone [illegible] ; genomic DNA; rearranged configuration; IgHeavy; regular; functionality unproductive; group IGHV.
[illegible]	[illegible]
