# Supplementary material for: IgH gene rearrangements as plasma biomarkers in Non-Hodgkin's Lymphoma patients
Source: Oncotarget. 2011 Mar 8;2(3):178–85. doi: 10.18632/oncotarget.235 (PMC3260819; doi:10.18632/oncotarget.235)
Supplement: Supplementary file 2 [file oncotarget-02-178-s002.docx]

Supplementary Table 2. Patientient characteristics

ID	Patienthology type	Sample types analyzed
Patient 1	Diffuse large B-cell lymphoma	Plasma
Patient 2	Diffuse large B-cell lymphoma	Plasma
Patient 3	Diffuse large B-cell lymphoma	Plasma
Patient 4	Diffuse large B-cell lymphoma	Plasma
Patient 5	Diffuse large B-cell lymphoma	Plasma
Patient 6	Diffuse large B-cell lymphoma	Plasma
Patient 7	High grade B-cell lymphoma not otherwise specified	Plasma
Patient 8	Burkitt's lymphoma	Plasma
Patient 9	Diffuse large B-cell lymphoma	Plasma
Patient 10	Diffuse large B-cell lymphoma	Plasma
Patient 11	Diffuse large B-cell lymphoma	Plasma
Patient 12	Diffuse large B-cell lymphoma	Plasma
Patient 13	Diffuse large B-cell lymphoma	Plasma
Patient 14	Diffuse large B-cell lymphoma	Plasma
Patient 15	B-cell lymphoma	Plasma
Patient 16	B-cell lymphoma	Primary tumor and plasma
Patient 17	B-cell lymphoma	Primary tumor and plasma
Patient 18	B-cell lymphoma	Primary tumor and plasma
