# Supplementary material for: IgH gene rearrangements as plasma biomarkers in Non-Hodgkin's Lymphoma patients
Source: Oncotarget. 2011 Mar 8;2(3):178–85. doi: 10.18632/oncotarget.235 (PMC3260819; doi:10.18632/oncotarget.235)
Supplement: Supplementary file 3 [file oncotarget-02-178-s003.docx]

Supplementary Table 3: Primers used for the validate of rearrangments by PCR*.			
**Patient ID**	**Primer1**	**Primer2**	**Primer1 position**
Patient 1	CTGGGTGGATTCTGAACAGC	GACGGTGACCAGGGTTCC	10541797
Patient 2	GCAGACACGGCTGTGTATTACT	CCCAGACGTCCATACCGTAG	10594867
Patient 3	GCGGACACGGCCATATATTA	CCCAGGAGTCGAACCAGT	N/A
Patient 4	CGGACACGGCCATCTATTAC	CCAGGGGTCGAACCAGTTTA	10554916
Patient 5	CCTGTGGACACAGCCACATA	CCAGTAGCCAAGTTCCATGC	10556519
Patient 6	TGGTATTACGATTCTTTGACTGG	CCTGAGGAGACGGTGACCA	10544159
Patient 7	CCAGGTGTGGTTATTGTCAGG	GGCCCCAGGTCAAAGTAGTA	10542084
Patient 8	CAGGCCGTGGTAGAGATGG	GTGACCAGGGTTCCCTGG	10542081
Patient 15	GCAGACACGGCTGTGTATTTT	TCACCTGAGGAGACGGTGAT	10594867
Patient 15	TGAGGTCTGTGTCACTGTGATATT	GAGACGGTGACCAGGGTTC	10544142
Patient 16	TTTTGATCGAGGAGACAGCA	CACCTGAGGAGACGGTGAC	10544129
Patient 16	CCAGGTGTGGTTATTGTCAGG	GCCATCTTACCTGAAGAGACG	10543961
Patient 17	AGGGACAGGAGGATTTTGTG	GCCCCAGTGGTGGAAGTATT	10542550
Patient 17	ACCTGAGAGTCGAGGACACG	TCACCTGAGGAGACGGTGAC	10611973

* In patients 9 to 10, the putative rearrangements identified by IgCap could not be confirmed by PCR. In patients 11 to 14, no rearrangements w
