# Supplementary material for: IgH gene rearrangements as plasma biomarkers in Non-Hodgkin's Lymphoma patients
Source: Oncotarget. 2011 Mar 8;2(3):178–85. doi: 10.18632/oncotarget.235 (PMC3260819; doi:10.18632/oncotarget.235)
Supplement: Supplementary file 4 [file oncotarget-02-178-s004.docx]

Supplementary Table 4:Primers used to construct IgH chip

Gene Name	Forward primer	Reverse primer	Size, bp
IGHV1-2	AGCACAGCTGCCTCCTCCCTT	GGAGATCTCATCCACTTCTGTGTT	402
IGHV1-3	CCAGCACAGCTGCTTCCC	TCCTGTGTCTTCTCTACAGGTGT	220
IGHV1-8	TTCTCCCCTAGGGTTTCTGACT	TCAGTCAAAGAGGATTTTATTCT	400
IGHV1-18	CTCCTCCCTGGGGTTTCTGAA	CCCCAGTCCCAGGGCTGAGA	431
IGHV1-24	TCATTCCTCAGGGTTTCTGACG	CTCCACAGGCACCCACGCC	365
IGHV1-45	CTCCTTGGGGTTTCTGACGG	CTTTTATCCACTCCTGTGTCCC	384
IGHV1-46	TGACACTCTGAGGATGTGGTTTC	GTGTCCTCTCCGCAGGTGCT	355
IGHV1-58	TACAGCTGCCTCCTTCCTGGC	GCTTTCATCCACTCCTGTGTT	397
IGHV1-69	TTCTCCCTCAGGGTTTCTGACA	CCTCTCCACAGGTGTCCAGTC	368
IGHV1-f	ATTCCTCAGGGTTTCTGACGC	CCTCTGTCCTCTCCACAGGCA	373
IGHV2-5	GGGTTTTTGTACAGGAGGTGC	CTTTCTCCACAGGGGTCTTG	364
IGHV2-26	AGCACCACTGAGATGCAGCTA	TTCCTCAGAAGTGAAACATCAGTTG	437
IGHV2-70	GGGTTCTTGTACAGGAAGCGC	TATGTCTTCTCCACAGGGGTCTTA	368
IGHV3-7	CCCCCTGGTGGTCCCAAG	ATTGAGTGTGTGTGGATATGAGTGT	459
IGHV(II)-20-1	TGTCTGGGCTCACACTGATG	ATCACAACCAGTGCTTCCTGA	569
IGHV3-9	TGTTTTCAGTGTGTCTTGAGTGC	GTGTGGCAGTTTCTGACCTT	447
IGHV3-11	CCCCCTGGTGGTCCCAA	CAGTGAATATATGTGGCAGTTTCTGACT	428
IGHV3-13	CAGCTGATTTCCCCCAGGCA	GGGTATGTGTGGCAATTTCTGACTTT	419
IGHV3-15	CCCGCCCTCAGTGGGTCA	GTGGATATGTGTGGCAGTTTCTGAT	455
IGHV3-20	TGTGTCTGGGCTCTCACTGG	AGAGATGTTGAGTGTGAGTGAACAC	427
IGHV3-21	GCACCTGCCCTGGAGACAGA	TGTGTGTGACAGTTTCTGACCAA	458
IGHV3-23	GGGAGGTTTGTGTCTGGGCT	GAGAAATAGAAAAATTGAGTGTGAA	443
IGHV3-33	CTGAGCGCCCCCTGCA	CTGAGACTCTCCTGTGCAGCG	330
IGHV3-43	CCTCCTGTCTTCCTGCAGC	CAGTGGATATGTGTGGCAGTG	418
IGHV3-48	CCCCCTGGTGGTCCTGC	GATATGAGTGAGAGAAACAGTGGATA	444
IGHV3-49	CCCCCTGGTGGCTCTGT	TGTGAGGGGACATGAGTGGT	458
IGHV3-53	CTGCCGCTGATTTCCCC	ATGTGTGGCAGTTTCTAACCAA	417
IGHV3-64	CGCCCCCTGCAGATGAC	GAGTGTGAGTGGACATGAGTGA	463
IGHV3-66	CCGCTGATTCCCCCAA	ATGTGTGGCAGTTTCTAACCAA	412
IGHV3-72	AGAGAAACAGTGGATTTGTGTGA	GTCCTCAGTGGTTCCCGAC	461
IGHV3-73	CAGTGGACGTGTGTGGCAC	TGTCCCGCCCTCAGTGA	460
IGHV3-74	ATGAGTGAGATAAGCAGTGGATG	GAGAGCAGCCCAGTTCAC	472
IGHV3-d	TGAGTGAGCAAAACAGTGGG	TTCCCCTCTGTGTATATCTGGC	377
IGHV4-4	CCAGGGCTCACCGTGGT	GCCCCCTCTGCCTATCTGT	402
IGHV4-28	GATATGGGAGGTGCCTCTGAG	AGCACCTGCGCCGGCT	436
IGHV4-30-2	TGATCCCAGGGCTCACTGTGT	CTCACACTCACCTCCCCTCAT	372
IGHV4-30-4	AGGGATCCAGACATGGGGG	GTCCCCTCCGCGTCCA	444
IGHV4-31	CAGGGCTCACTGTGGGTTTT	CCTGCTGGCCCTGAGCT	433
IGHV4-34	CAGGGCTCACTGTGGGTT	CCTGCTGGTCTTGAGCAGT	427
IGHV4-61	GCAGCTCCCAGATGTGAGTA	TCCCTGCACGGAGGTTTT	450
IGHV5-51	TGATTCAAATTTTGTGTCTCCCC	GGCTCGGGGCTGGTTTC	362
IGHV6-1	GTGTCAGGGAGATGCCGTAT	AGGTTTGTGTCTGGGCTCAC	435
IGHV7-4-1	AGGCAGAAACCAGGCCAC	CACAGCTGCCTCCCACA	424
IGHJ1	GCTATCCCCAGACAGCAGAC	TGAGCTGAGAACCACTGTGC	198
IGHJ2	AGGGATGGCAGCTGGTGT	GCAGAGGCTGTGCTACTGGT	177
IGHJ3	CTCCCAGCTCCAGGACAGAG	GTGACCCATTCGAGTGTCCT	184
IGHJ4	CAGGGCATCCTCCTGAGC	GGCCTCCCACAATGTGACTA	195
IGHJ5	TTTCCTGACCTCCAAAATGC	GGTCCTGGCATTGTTGTCAC	175
